# Deep Classification with Linearity-Enhanced Logits to Softmax Function

**DOI:** 10.3390/e25050727

**Published:** 2023-04-27

**Authors:** Hao Shao, Shunfang Wang

**Affiliations:** 1 School of Mathematics and Statistics, Yunnan Unverisity, Kunming 650504, China; 2 School of Information Science and Engineering, Yunnan Unverisity, Kunming 650504, China; 3 The Key Lab of Intelligent Systems and Computing of Yunnan Province, Yunnan University, Kunming 650504, China

**Keywords:** convolutional neural network, classification, Orthogonal Softmax, Gram–Schmidt orthogonalization

## Abstract

Recently, there has been a rapid increase in deep classification tasks, such as image recognition and target detection. As one of the most crucial components in Convolutional Neural Network (CNN) architectures, softmax arguably encourages CNN to achieve better performance in image recognition. Under this scheme, we present a conceptually intuitive learning objection function: Orthogonal-Softmax. The primary property of the loss function is to use a linear approximation model that is designed by Gram–Schmidt orthogonalization. Firstly, compared with the traditional softmax and Taylor-Softmax, Orthogonal-Softmax has a stronger relationship through orthogonal polynomials expansion. Secondly, a new loss function is advanced to acquire highly discriminative features for classification tasks. At last, we present a linear softmax loss to further promote the intra-class compactness and inter-class discrepancy simultaneously. The results of the widespread experimental discussion on four benchmark datasets manifest the validity of the presented method. Besides, we want to explore the non-ground truth samples in the future.

## 1. Introduction

In the past few years of artificial intelligence research, Convolutional Neural Networks (CNNs) have played a crucial role in deep learning classification tasks. Benefiting from advanced network architecture [1,2,3,4,5,6,7,8,9,10,11,12,13,14,15,16] and discriminative capacity [17], CNNs have dramatically upgraded the performance across various visual classification tasks, such as object recognition [18,19], face verification [20,21], molecular biology [22,23], and hand-written digit recognition [24]. A recent current towards learning is to strengthen CNN with more discriminative power and more applied scenarios. In the research of machine learning, Long [25] proposed a new self-training semi-supervised deep learning (SSDL) method to further explore the fault diagnosis models. Xu [26] presented the Global Contextual Multiscale Fusion Network (GCMFN) to better accommodate noisy and unbalanced scenarios. In addition, several studies have employed CNN in the medical field. Fan [27] attached SVM to the fully connected layer to better identify the cancer datasets. Sekhar [28] proposed a novel transfer learning method to detect brain tumors. As arguably one of the most crucially used components in CNN architecture, softmax is widely used in image classification tasks.

Intuitively, the softmax loss is a popular choice to learn discriminative features in the pioneering work [29], but the original softmax loss only discriminates between partial features and does not separate inter-class features enough. Several variants have been offered to enhance the discriminative capacity of the softmax loss. The center loss [30] was proposed to compact the intra-class distance by calculating the L2 distance between the feature vector and its class center. By the cooperative penalization of the softmax, center loss achieves stronger discriminability and obtains a smaller intra-class distance. However, updating the factual center is impractical as the training number grows.

Some research has also adapted distance constraints to a pair or a triplet of samples to improve the discriminative power so that similar samples are as compact as possible and dissimilar samples are as spread apart as possible. For example, contrastive loss [31] further distinguishes similar samples from different samples by feature extraction. On the other hand, regarding triplet loss, Refs. [32,33,34] presented the triplet training samples for the first time. This method guarantees an anchor sample is far from a negative sample and is close to a positive sample in a triplet of samples. Furthermore, batches of all triplet loss [35] and hard triplet loss [36] were proposed to administer more constraints and achieved a stronger generalization capability of features. Based on triplet loss, N-pair loss [37] employs a positive sample and multiple negative samples for an anchor sample to train the network. Specifically, N-pair loss applies N-1 negative samples in each training phase, which selects more information and increases the convergence speed. However, both contrastive loss and triplet loss cannot oblige on each sample of features, which will lead to unstable convergence as the size of the samples grows dramatically. Although these methods can achieve higher discriminative power and diminish this problem, they also complicate the network and make training more difficult.

On the other hand, various studies have attempted to reformulate the softmax by implementing the margin-based loss function. Unlike previous loss methods, these studies aim to improve the discriminability of softmax loss by presenting the angular penalty, which was set between feature vectors and corresponding weight vectors of the last fully connected layer. By the angular margin *m*, the margin-based loss functions enhance inter-class distance and try to achieve stronger discriminability. For instance, SphereFace [38,39] and L-Softmax [40] first came up with the concept of angular margins by a multiplicative angular penalty, which further separated various classes and compacted the same classes. The novel loss functions enhance the discriminability of features by the change of the decision boundary, but these can lead to an unstable training process due to the difficulty of optimization. CosFace [41], AM-Softmax [42], and Soft-Softmax [43] suggested enhancing angular discriminative power by use of an additive cosine margin. Benefitting from the cosine margin, this can thereby further develop the discriminative power and provide an intuitive explanation. Building on the previous method, ArcFace [44,45] presented an additive angular margin that effectively unites the multiplicative angular margin, cosine margin, and angular margin. Profiting from the advantages of a unified framework, ArcFace plays a crucial role in deep classification and achieved sample features with stronger discriminability. For the ArcFace, the feature margin between different classes was set to the same and fixed, and this may not adapt to the real situation of various classes. In addition, several lines of research have been improved in various directions based on ArcFace. For example, Dyn-Arcface [46] replaced the flexible margin penalty based on the distance between each class center and the other class centers. ElasticFace loss [47] relaxes the fixed single margin by deploying a random margin drawn from a normal distribution. To reflect the more real properties of class separability, Groupface [48] suggested enriching the feature representations with group-aware representations based on the Arcface. AdaptiveFace put forward hard prototype mining (HPM) to adaptively adjust the margins between various classes to solve the problem of imbalanced training data in deep classification. Moreover, Uniformface [49] presented equalized distances between various class centers by adding a new loss function on SphereFace. ASL [50] mitigates the bias induced by data imbalance and increases interclass diversity. It is obvious that the flexible models present better performance compared to the fixed margin. Generally, the margin-based loss functions enhance the inter-class discrepancy by proposing an angular penalty, which is between feature vectors and the corresponding weight vectors of the last fully connected layer. However, these methods only penalize the partial samples in the angular space, which leads to unfair considerations for every class.

Based on this, we present a linear Orthogonal-Softmax loss to achieve stronger discriminability. Inspired by the Taylor-Softmax [51], the proposed Orthogonal-Softmax presents various orthogonal polynomials approximation for the $e^{z}$ of softmax, which is designed by executing Gram–Schmidt orthogonalization. By employing an approximated linear logit, the proposed Orthogonal-Softmax has a sturdier linear relationship than the softmax loss and the Taylor-Softmax. On the other hand, benefitting from the thinking of CosFace and AM-Softmax, we added margin *m* to the new loss and achieved Orthogonal-M. Compared to the Orthogonal-Softmax, Orthogonal-M increases the inter-class separation and achieves stronger discriminative power. The principal contributions can be outlined as follows:

(1) The proposed Orthogonal-Softmax applies Gram–Schmidt orthogonalization in the softmax loss, which presents the approximated orthogonal polynomials for the exponential function of softmax. Additionally, in order to verify the fitting effect of the new loss functions, we compare various series of orthogonal polynomials to the Taylor series.

(2) In order to achieve the stronger discriminative power, we employ the idea of inter-class margin *m* to the Orthogonal-Softmax and obtain Orthogonal-M. The proposed Orthogonal has a better geometric attribute, which enhances inter-class discrepancy and intra-class compactness.

(3) Extensive experiments are conducted on four benchmark datasets (MNIST, Fashion-MNIST, CIFAR10, and CIFAR100). The results demonstrate the effectiveness of the Orthogonal-Softmax and Orthogonal-M, which have better performances over the Taylor-Softmax and softmax loss.

## 2. Related Work

In recent years, the softmax loss has been widely used as a key method to learn discriminative features for multiclass classification. Several margin-based methods have been presented to enhance the discriminative power of the softmax loss. These studies have added a margin penalty into various classes to create inter-class feature separability. SphereFace [38,39], CosFace [41], AM-Softmax [42], and ArcFace [44,45] all introduce an additive angular margin between the features and their corresponding weights under various manners. On the other hand, Taylor-Softmax [51], LinCos-Softmax [52] and LinArc [53] have proposed an approximated linear model, which creates a stricter relationship by Taylor expansion.

In addition, margin-based softmax loss functions enforce better intra-class compactness and inter-class diversity, but these studies have not effectively emphasized every sample according to its practical importance. To a certain extent, Taylor approximated softmax loss enhances the linear relationship with the angle, but they may not have enough discriminative power. Based on margin-based softmax loss and approximated linear softmax loss, we introduce a novel loss function through Gram–Schmidt orthogonalization. By combining the strengths of both, Orthogonal-Softmax has a better approximate effect and enhances the discriminative power through experiments on four datasets.

## 3. Overview of the Proposed Method

In this section, we will introduce the relative definition and derivation of orthogonal polynomials first. Based on the previous definition, then we will present the proposed Orthogonal-Softmax and the whole process of the Gram–Schmidt orthogonalization algorithm.

### 3.1. Introduction of Orthogonal Polynomials

Orthogonal polynomials are generally calculated by Gram–Schmidt orthogonalization, and we mainly introduce the idea of the nearest distance between orthogonal polynomials and the target function as follows:

Following the definition of Axler [54]: *U* is a subspace of the inner product *V*, for any vector v∈V, and we have:

(1) $$∥v-P_{U}v∥\;\leq \;∥v-u∥$$

and

(2) $$P_{U}v=\langle v_{j},e_{1}\rangle e_{1}+\cdots +\langle v_{j},e_{j-1}\rangle e_{j-1}$$

where $P_{U}v$ is the orthogonal projection on *U* and $e_{i}$ is the orthonormal basis of *v*.

Based on the definition, it can be inferred that $P_{U}v$ is the shortest distance from *v* to *V*. In this way, we assume that the vector *v* is the exponential function in the softmax, and the orthogonal polynomial is the $P_{U}v$. Finding the shortest distance from subspace *V* to vector *v* means finding the best approximated orthogonal polynomial for the exponential function *e* in the inner product space.

### 3.2. Orthogonal-Softmax

In more detail, the softmax loss is defined by the formula:

(3) $$L_{Softmax}=\frac{e^{z_{i}}}{\sum_{j=1}^{K}e^{z_{j}}}\;\;\;for\;i=1,\cdots ,K$$

where $z=\left(z_{1},\cdots \;z_{K}\right)\in R^{K}$ and $z_{i}$ denotes the deep feature of the *i*th input vector **z**, and $y_{i}$ is the corresponding class. *K* is the sum of the classes.

In order to enhance the discriminability of features and produce an excellent result, we designed a linear approximated logit by applying the orthogonal polynomials. The Orthogonal-Softmax loss can be defined as:

(4) $$L_{Orthogonal-Softmax}=\frac{f^{n}\left(z\right)}{\sum_{j=1}^{K}f^{n}\left(z\right)}$$

where

(5) $$f^{n}\left(z\right)=\sum_{i=0}^{n}\langle v,e_{i}\rangle e_{i}$$

and

(6) $$e_{i}=\frac{\beta_{i}}{∥\beta_{i}∥}$$

and

(7) $$\beta_{i}=\alpha_{i}-\frac{\langle \alpha_{i},\beta_{1}\rangle }{\langle \beta_{1},\beta_{1}\rangle }\beta_{1}-\cdots -\frac{\langle \alpha_{i},\beta_{i-1}\rangle }{\langle \beta_{i-1},\beta_{i-1}\rangle }\beta_{i-1},$$

where $\alpha_{i}$ is a linearly independent list of vectors in inner-product space *V*, and we can apply the Gram–Schmidt procedure to the $\alpha_{i}$ to obtain the orthogonal basis $\beta_{i}$ and orthonormal basis $e_{i}$. Furthermore, *U* is the subspace of *V* where v∈V and $P_{U}v=\langle v,e_{1}\rangle e_{1}+\cdots +\langle v,e_{i}\rangle e_{i}$ is the orthographic projection on *U* to *V*. Generally, the orthographic projection is the shortest distance from *V* to *U*, and we can denote $P_{U}v$ as the best approximation, so it is the smallest distance to the exponential function; hence, we can use the orthogonal polynomial to approximate the softmax.

We can present the whole process of the second-order approximated logit, thus a group of basis was given by:

(8) $$\alpha_{0}=1,\alpha_{1}=x,\alpha_{2}=x^{2}$$

i=0:

(9) $$\alpha_{0}=1,\beta_{0}=\alpha_{0}=1,e_{0}=\frac{\beta_{0}}{∥\beta_{0}∥}=1$$

i=1:

(10) $$\begin{array}{c} \begin{array}{cc} \alpha_{1} & =x,\\ \beta_{1} & =\alpha_{1}-\frac{\langle \alpha_{1},\beta_{0}\rangle }{\langle \beta_{0},\beta_{0}\rangle }\beta_{0}=x-\frac{1}{2},\\ e_{1} & =\frac{\beta_{1}}{∥\beta_{1}∥}=2\sqrt{3}\left(x-\frac{1}{2}\right) \end{array} \end{array}$$

i=2:

(11) $$\begin{array}{c} \begin{array}{cc} \alpha_{2} & =x^{2},\\ \beta_{2} & =\alpha_{2}-\frac{\langle \alpha_{2},\beta_{0}\rangle }{\langle \beta_{0},\beta_{0}\rangle }\beta_{0}-\frac{\langle \alpha_{2},\beta_{1}\rangle }{\langle \beta_{1},\beta_{1}\rangle }\beta_{1}=x^{2}-x+\frac{1}{6}\\ e_{2} & =\frac{\beta_{2}}{∥\beta_{2}∥}=6\sqrt{5}\left(x^{2}-x+\frac{1}{6}\right), \end{array} \end{array}$$

where after a simple and intuitive calculation, an approximated linear polynomial can be presented as:

(12) $$\begin{array}{c} \begin{array}{cc} f^{n}\left(z\right) & =\langle v,e_{0}\rangle e_{0}+\langle v,e_{1}\rangle e_{1}+\langle v,e_{2}\rangle e_{2}\\ \; & =0.839x^{2}+0.851x+1.012993 \end{array} \end{array}$$

The second-order orthogonal polynomials approximation for softmax has been proved by this, and the whole process of Gram–Schmidt Orthogonalization can be calculated in this way. The various series of the orthogonal basis $\beta_{i}\left(x\right)$ and orthonormal basis $e_{i}\left(x\right)$ are presented in Table 1. Various series of orthogonal polynomials $f_{i}\left(x\right)$ will be directly presented in the classification experiments of Section 3.

### 3.3. Comparing with Taylor-Softmax

By using Taylor series approximation, the Taylor-Softmax [39] can be defined as:

(13) $$L_{Taylor-Softmax}=\frac{f^{n}\left(z\right)}{\sum_{j=1}^{K}f^{n}\left(z\right)}$$

where

(14) $$f^{n}\left(z\right)=\sum_{i=0}^{n}\frac{z^{i}}{i!}\;for\;i=1,2,\cdots ,K$$

**Figure 1:**
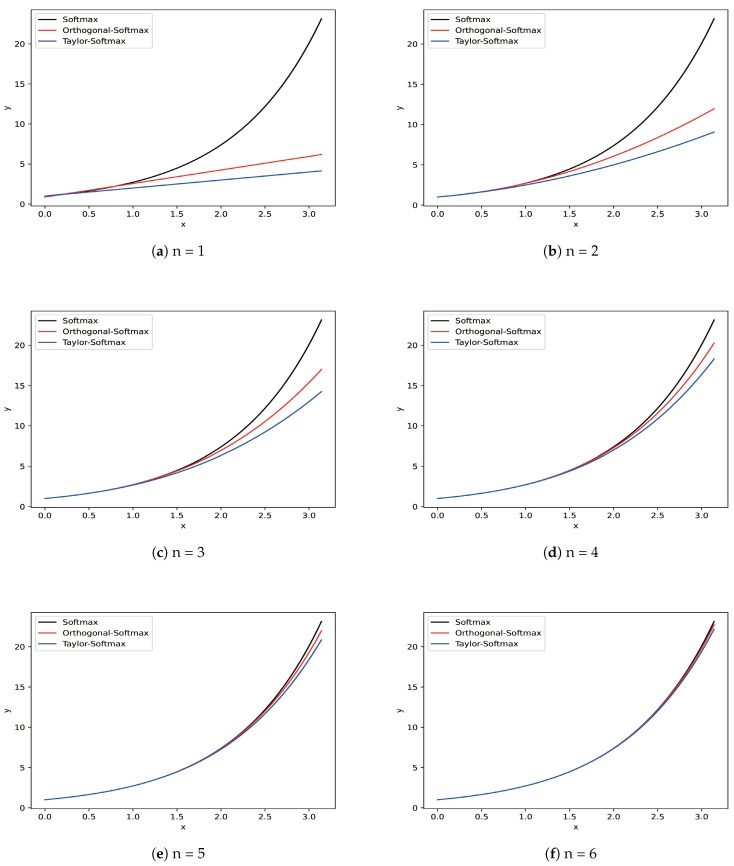
Curves for comparing the approximate effects of Orthogonal-Softmax and Taylor-Softmax on various series. The Orthogonal Softmax presents a preferable approximation on all six series.

The linear approximated effects of orthogonal polynomials and Taylor polynomials on various series are shown in Figure 1. Black lines denote the exponential functions of softmax, red lines denote the orthogonal polynomials of Orthogonal-Softmax, and blue lines denote the Taylor polynomials of Taylor-Softmax. Compared to Taylor polynomials, the orthogonal polynomials present the better approximation for the exponential functions on all series. In addition, as the series increases, both polynomials achieve stronger approximated effects, which we will demonstrate further in the following experiments.

Back to Equation (3), when dealing with the binary-classes scenarios with the *class* 1 and *class* 2, the original Softmax loss presents a decision boundary by $z_{1}=z_{2}$. To make the intra-class more compact, CosFace [41] and AM-Softmax [42] introduce an additive margin *m* and the decision boundary given by:

(15) $$\begin{array}{cc} class\;1:z_{1}\;>\; & z_{1}-m>z_{2}\\ class\;2:z_{2}\;>\; & z_{2}-m>z_{1} \end{array}$$

where *m* is a fixed parameter presented to control the margin of inter-class. By introducing $z_{1}-m$ to relace $z_{1}$, the logits can be defined as:

(16) $$L_{AM-Softmax}=\frac{e^{z_{i}-m}}{e^{z_{i}-m}+\sum_{j=1}^{K}e^{z_{j}}}\;\;\;for\;i=1,2,\cdots ,K.$$

**Figure 2:**
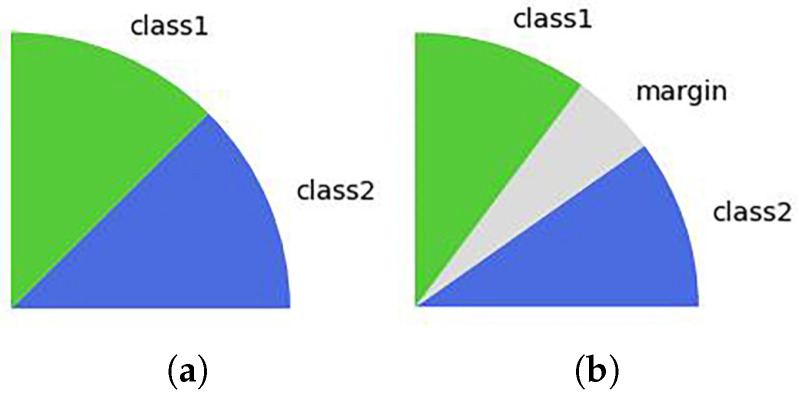
The comparison of decision boundary on the binary class scenarios. Class 1 and class 2 are represented by green and blue zones, respectively, and gray areas are design margins. As shown in (**b**), *m* is an additive margin to further increase inter-class distance. (**a**) Orthogonal-Softmax; (**b**) Orthogonal-M.

Based on the thinking of AM-Softmax, we also introduced margin *m* into Orthogonal Softmax and Taylor-Softmax as Orthogonal-M and Taylor-M, respectively. As illustrated in Figure 2, by employing an additive margin, the proposed Orthogonal-M enhances inter-class discrepancy and intra-class compactness.

## 4. Experiments

### 4.1. Implementation Details

In the following experiment, we employ MNIST, FashionMNIST, CIFAR10, and CIFAR100 as our training datasets in order to create a fair comparison with Taylor-Softmax [39]. It is important to note that $f^{n}\left(z\right)$ is definite for n=2k in Taylor-Softmax, so we will look into the orthogonal polynomials series expansion of 2, 4, and 6 on the experiment design method for the convenience of comparison. Our aim was not to achieve the best accuracy for these datasets but to explore new ways to enhance the discriminative power of Softmax.

### 4.2. Experimental Setting

As shown in Table 2, we present the network structure corresponding to each dataset. During the experiment process, we evaluate the generalized softmax loss in visual classification. For the CNN construction, we adopt the VGG-net, as well as the Taylor-Softmax for convenient comparison. During all experiments, we assume the PRelu as the activation and the batch size is 256. Convolution neural network training is finished with SGD with momentum 0.9 and weight decay 0.0005.

**Figure 3:**
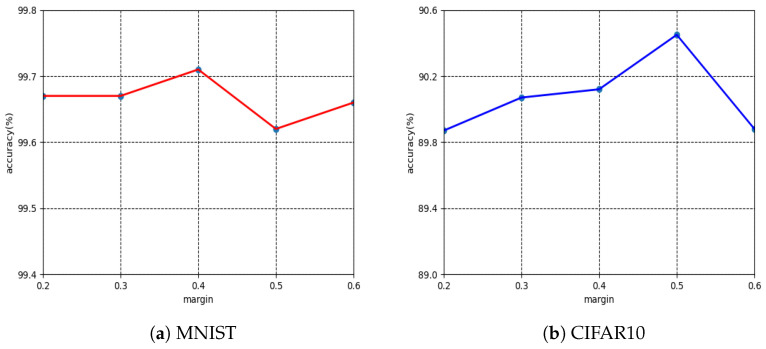
Accuracy (%) of Orthogonal-M with various margin parameters *m* on MNIST and CIFAR10 under sixth-order orthogonal series approximation.

The margin parameter *m* plays a crucial role in the proposed the Orthogonal-M. Following the setup of the previous work, we varied margin *m* from 0 to 0.6 with a deep size of 0.1, which was set in both Taylor-M and Orthogonal-M. As shown in Figure 3, for the sixth-order orthogonal series of the proposed Orthogonal-M, MNIST and CIFAR10 achieved the highest accuracy levels at m=0.4 and m=0.5, respectively. This indicates that different datasets have various intrinsic properties, and it may not be effective to treat all datasets with the same fixed parameter *m*. Thus, we present the best accuracy levels of each dataset in various *m* values. In all these topologies, we replace the final Softmax function with each of its alternatives in our experiments.

### 4.3. Evaluation Results

The experimental results shown in Table 3 are the verification accuracies on four datasets under various methods.

As shown in Table 3, we display the performance of our proposed Orthogonal-Softmax with series variables 2 to 6 to correspond to Taylor-Softmax. The bold numbers in each column are the highest verification result for various models on each dataset. From the quantitative comparison among all the methods on four datasets, Orthogonal-Softmax presents better performance than Softmax loss and Taylor-Softmax. Benefitting from the introduction of margin-based loss functions, the proposed Orthogonal-M further enhances the discriminative power based on the Orthogonal-Softmax. For the MNIST and Fashion-MNIST, it is well known that these two datasets are simple and typical in deep classification tasks, so the results in the proposed methods are not significantly improved. On CIFAR10, the proposed Orthogonal-M achieves an improvement of 90.45%, which is 3.58% over the Softmax loss and 3.28% over the Taylor-Softmax. Additionally, on CIFAR100, the proposed Orthogonal-M is 8.45% higher than the Softmax loss and 7.08% higher than the Taylor-Softmax. It is worth noting that the proposed Orthogonal-M partially achieves lower performance on series 2 and 4 when compared with Orthogonal-Softmax. The reason for this is that series 6 has a better approximation effect and is more steady, and the added margin presents a better reflection of margin-based Softmax. As observed, all the verification experiments show that the proposed linear logits can appropriately distinguish features and outperform the other existing methods.

## 5. Conclusions

In this paper, we proposed the Orthogonal-Softmax loss function, which employs an approximated linear logit to effectively replace the Softmax loss. By taking Gram–Schmidt orthogonalization, the proposed Orthogonal-Softmax was able to achieve a better linear relationship and stronger discriminative power. We also supplied a mathematical explanation to dissect the whole process of Gram–Schmidt orthogonalization and its advantages. Experimental results and analyses on four well-known benchmark datasets (MNIST, Fashion-MNIST, CIFAR10, and CIFAR100) demonstrate the superiority of the proposed Orthogonal-Softmax and Orthogonal-M when compared to other loss functions. Therefore, how the conundrum of non-ground-truth classes can be resolved, as well as how bigger datasets can be explored will be our future work.

## Figures and Tables

**Table 1:** The presented Orthogonal Basis $\beta_{i}\left(x\right)$ and Orthonormal Basis $e_{i}\left(x\right)$ for various series *i*.

	Function	$\beta_{i}\left(x\right)$	$e_{i}\left(x\right)$
Series			
i=0		1	1
i=1		$x-\frac{1}{2}$	$2\sqrt{3}\left(x-\frac{1}{2}\right)$
i=2		$x^{2}-x+\frac{1}{6}$	$6\sqrt{5}\left(x^{2}-x+\frac{1}{6}\right)$
i=3		$x^{3}-\frac{2}{3}x^{2}+\frac{3}{5}x-\frac{1}{20}$	$20\sqrt{7}\left(x^{3}-\frac{2}{3}x^{2}+\frac{3}{5}x-\frac{1}{20}\right)$
i=4		$x^{4}-2x^{3}+\frac{9}{7}-\frac{2}{7}x+\frac{1}{70}$	$210\left(x^{4}-2x^{3}+\frac{9}{7}-\frac{2}{7}x+\frac{1}{70}\right)$
i=5		$x^{5}-\frac{5}{2}+\frac{20}{9}x^{3}-\frac{5}{6}x^{2}+\frac{5}{42}x-\frac{1}{252}$	$253\sqrt{11}\left(x^{5}-\frac{5}{2}+\frac{20}{9}x^{3}-\frac{5}{6}x^{2}+\frac{5}{42}x-\frac{1}{252}\right)$
i=6		$x^{6}-3x^{5}+\frac{75}{22}x^{4}-\frac{20}{11}x^{3}+\frac{5}{11}x^{2}-\frac{1}{22}x+\frac{1}{924}$	$924\sqrt{13}\left(x^{6}-3x^{5}+\frac{75}{22}x^{4}-\frac{20}{11}x^{3}+\frac{5}{11}x^{2}-\frac{1}{22}x+\frac{1}{924}\right)$

**Table 2:** Topologies of four various datasets.

	Datasets	MNIST	Fashion-MNIST	CIFAR10	CIFAR100
Layer					
Conv0.X		[3×3,64] ×1	[3 × 3,64] ×1	[3 × 3,64] ×1	[3 × 3,96] ×1
Conv1.X		[3 × 3,64] ×3	[3 × 3,64] ×3	[3 × 3,64] ×4	[3 × 3,96] ×4
Pool1		2 × 2 Max,Stride 2	2 × 2 Max,Stride 2	2 × 2 Max,Stride 2	2 × 2 Max,Stride 2
Conv2.X		[3 × 3,64] ×3	[3 × 3,64] ×3	[3 × 3,96] ×4	[3 × 3,128] ×4
Pool2		2 × 2 Max,Stride 2	2 × 2 Max,Stride 2	2 × 2 Max,Stride 2	2 × 2 Max,Stride 2
Conv3.X		[3 × 3,64] ×3	[3 × 3,96] ×3	[3 × 3,128] ×4	[3 × 3,384] ×4
Pool3		2 × 2 Max,Stride 2	2 × 2 Max,Stride 2	2 × 2 Max,Stride 2	2 × 2 Max,Stride 1
Fully Connected		256	256	256	512
Fully Connected		10	10	10	100

Note: Conv1.X, Conv2.X, and Conv3.X indicate convolution units that may contain multiple convolution layers. The [3,3,96] ×3 indicates three cascaded convolution layers with 96 filters of size 3 × 3.

**Table 3:** Verification results (%) of different loss functions.

Dataset	Variants	Accuracy	2	4	6
MNIST	softmax	99.41%			
	Taylor	99.59%	99.54%	99.59%	99.50%
	Taylor-M	99.67%	99.67%	99.59%	99.53%
	Orthogonal	99.68%	99.68%	99.64%	99.68%
	Orthogonal-M	**99.71%**	**99.69%**	**99.64%**	**99.71%**
Fashion-MNIST	softmax	93.55%			
	Taylor	93.92%	93.84%	93.71%	93.92%
	Taylor-M	93.98%	93.52%	93.23%	93.98%
	Orthogonal	94.40%	**94.40%**	94.30%	94.33%
	Orthogonal-M	**94.45%**	93.81%	**94.32%**	**94.45%**
CIFAR10	softmax	86.87%			
	Taylor	87.17%	86.86%	87.06%	87.17%
	Taylor-M	87.47%	87.47%	86.86%	87.08%
	Orthogonal	89.75%	**89.75%**	**89.72%**	89.55%
	Orthogonal-M	**90.45%**	89.69%	89.71%	**90.45%**
CIFAR100	softmax	48.57%			
	Taylor	49.94%	44.70%	49.24%	49.94%
	Taylor-M	49.95%	44.77%	49.95%	49.56%
	Orthogonal	56.67%	**54.53%**	56.50%	56.67%
	Orthogonal-M	**57.02%**	54.47%	**56.81%**	**57.02%**

## Data Availability

Not applicable.
